# A Normal Sensor Calibration Method Based on an Extended Kalman Filter for Robotic Drilling

**DOI:** 10.3390/s18103485

**Published:** 2018-10-16

**Authors:** Dongdong Chen, Peijiang Yuan, Tianmiao Wang, Ying Cai, Haiyang Tang

**Affiliations:** 1 School of Mechanical Engineering and Automation, Beihang University, Beijing 100191, China; itr@buaa.edu.cn (P.Y.); itm@buaa.edu.cn (T.W.); cy1001@buaa.edu.cn (Y.C.); 2 Shanghai Aircraft Manufacturing Co., Ltd., Shanghai 200436, China; tanghaiyang@comac.cc

**Keywords:** parameter identification, normal adjustment, extended Kalman filter, laser displacement sensor, robotic drilling system

## Abstract

To enhance the perpendicularity accuracy in the robotic drilling system, a normal sensor calibration method is proposed to identify the errors of the zero point and laser beam direction of laser displacement sensors simultaneously. The procedure of normal adjustment of the robotic drilling system is introduced firstly. Next the measurement model of the zero point and laser beam direction on a datum plane is constructed based on the principle of the distance measurement for laser displacement sensors. An extended Kalman filter algorithm is used to identify the sensor errors. Then the surface normal measurement and attitude adjustments are presented to ensure that the axis of the drill bit coincides with the normal at drilling point. Finally, simulations are conducted to study the performance of the proposed calibration method and experiments are carried out on a robotic drilling system. The simulation and experimental results show that the perpendicularity of the hole is within 0.2°. They also demonstrate that the proposed calibration method has high accuracy of parameter identification and lays a basis for high-precision perpendicularity accuracy of drilling in the robotic drilling system.

## 1. Introduction

In aircraft assembly, mechanical connections are the main type of connection between the components of the aircraft. The connection quality at the drilling hole has significant influence on the quality and lifespan of the aircraft [1,2,3]. With the traditional manual drilling method it has been difficult to meet the high-precision, high-efficiency and low-cost requirements of aircraft [4]. With the continuous development of robotics technology, robotic drilling systems will be the development trend in aircraft assembly [5]. The perpendicularity of the hole is an important factor affecting the quality of the hole, which is mainly determined by the angle between the normal at the drilling point and the axis of the drill bit. If the angle between the axis of the drill bit and the normal at drilling point is larger, the diameter of the hole becomes larger, the cutting force in the drilling process increases, the cutting heat increases, the burr height increases, and the life of the tool decreases [6]. In addition, it will affect the connection quality and fatigue strength of aircraft components [7]. For new fighter aircraft, this has an important effect on the performance at supersonic speeds and stealth. Therefore, it is of great significance to study the normal adjustment technique to improve the perpendicularity accuracy of the hole. The normal adjustment technique mainly includes two aspects: surface normal measurement and attitude adjustment [8]. At present, laser displacement sensors are widely used in the surface normal measurement of robotic drilling systems [9]. Laser displacement sensors are commonly used non-contact measurement sensors, which have many advantages such as high-precision, low power consumption, high reliability and security [10]. Typically, three or four laser displacement sensors are installed on the pressure foot for surface normal measurement [11,12]. Due to the machining error of the pressure foot and the assembly errors, there are always small errors between the nominal and actual zero points and laser beam directions of laser displacement sensors. These errors will lead to a decrease in the surface normal measurement accuracy. Therefore, it is crucial to study the parameter identification of laser ranging sensors to improve the surface normal measurement accuracy.

Yuan et al. proposed a surface normal measurement method with four laser displacement sensors [13,14]. Wang et al. developed a multifunctional automatic drilling end-effector with four laser displacement sensors to measure the surface normal at the drilling point [15]. However, the assembly errors of sensors aren’t considered in these two methods. Lu et al. proposed a method to calibrate the laser beam direction by using a coordinate measuring machine platform and a calibration block, with an adjustable plane. Bi et al. developed some calibration methods based on a coordinate measuring machine platform, in which a parameter substitution method [16] and non-linear least squares algorithm [17] are used to determine the zero point and laser beam direction. Both of the methods mentioned above can meet the accuracy requirement, and both of these methods require high-precision step motion with the same spacing on the coordinate measuring machine platform. However, industrial robots cannot achieve the high-precision requirement of step motion with the same spacing, and the solution process is too complicated. The rotation and translation matrices between the industrial robot and laser can be calculated by using a standard sphere [18]. Yuan et al. used the plane and sphere fitting methods to compute the zero point and laser beam direction on an industrial robot platform [19]. The two methods rely on the theoretical kinematics model of industrial robots, and the actual kinematic models are used in the experiment. However, since machining errors, assembly errors and load, the theoretical and actual kinematic models of industrial robots are different. Cao et al. proposed a calibration method based on math model and the least square method to obtain the zero point and laser beam direction [20]. However, in the calibration step of the method, the axis of drill bit should be adjusted so that it is perpendicular to the datum plane. This step requires a lot of time and the calibration procedure is complex and inefficient.

This paper proposes a normal sensor calibration method based on and Extended Kalman Filter (EKF) to identify the errors of zero points and laser beam direction for robotic drilling. Firstly, the procedure of normal adjustment of the robotic drilling system is introduced. Next, the measurement model of the zero point and laser beam direction on datum plane is constructed based on the principle of the distance measurement for laser displacement sensors. The EKF is used to identify the errors of zero points and laser beam direction of laser displacement sensors. Then the normal adjustment to achieve high-precision normal measurement and attitude adjustment is presented. Finally, simulations and experiments are conducted to demonstrate the correctness and validity of the proposed calibration method. The simulation and experimental results show that the proposed calibration can improve the perpendicularity accuracy of drilling and meet the accuracy requirement of aircraft assembly.

The rest of the paper is organized as follows: Section 2 develops the procedure of normal adjustment for the robotic drilling system. Section 3 derives the measurement model and applies the EKF algorithm to obtain the zero point and laser beam direction errors. Section 4 presents the normal measurement and attitude adjustment. Section 5 proposes the simulation study to verify the proposed method. Section 6 implements a calibration experiment on a robotic drilling system. The conclusions are proposed in the final section.

## 2. The Procedure of Normal Adjustment

**Figure 1:**
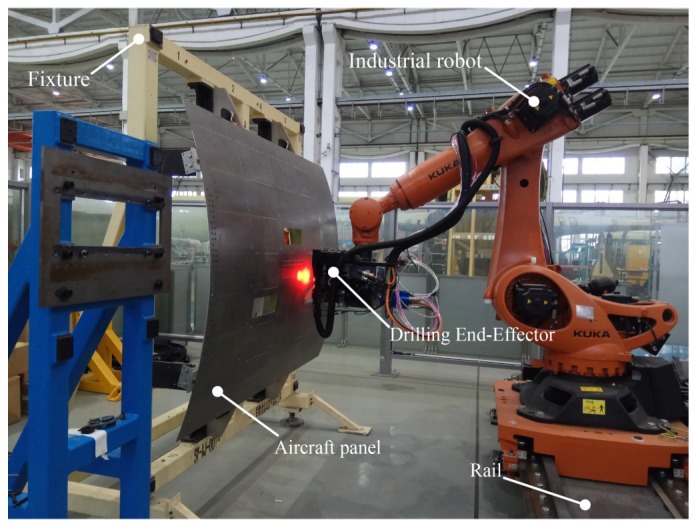
The robotic drilling system.

As shown in Figure 1, the robotic drilling system consists of an industrial robot, drilling end-effector, rail, fixture and aircraft panel. The drilling end-effector is attached to the flange of the industrial robot with six joints. The industrial robot can move along the rail for rectilinear motion. The aircraft panel is attached to a fixture to maintain stiffness during the drilling process. The robotic drilling system has various functions such as positional error compensation of robot, visual compensation, surface normal measurement, attitude adjustment, pressure foot clamping, drilling, scraping, micro-lubrication, etc. The robotic drilling system can meet the high-precision and high-efficiency requirements of automatic drilling.

**Figure 2:**
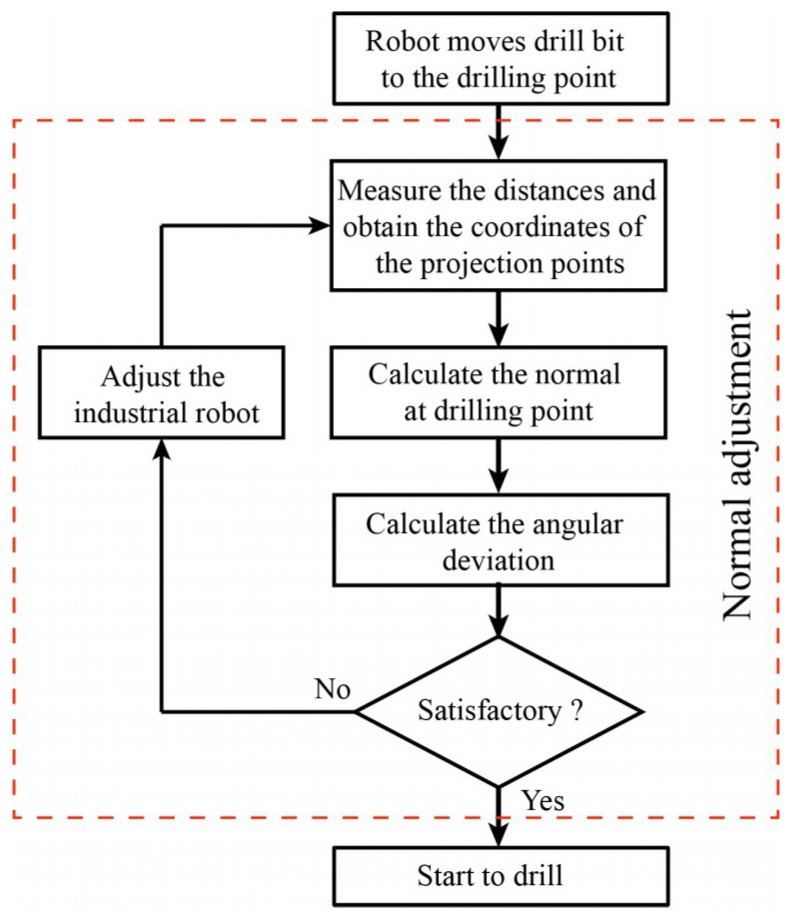
The flow of normal adjustment of robotic drilling system.

The normal adjustment is one of the crucial key technologies of the robotic drilling system and it has an important influence on the perpendicularity accuracy of holes. The normal adjustment mainly consists of surface normal measurement and attitude adjustment. The flow of surface normal adjustment for the robotic drilling system is depicted in Figure 2. The robot moves the drill bit to the drilling point according to the numerical control (NC) instructions. The distances between the sensor zero points and projection points can be measured by the laser displacement sensors and the coordinates of the projection points can be calculated. The normal at the drilling point can be computed by using surface normal measurement algorithm. If the angular deviation between the surface normal at drilling point and the axis of the drill bit does not meet the accuracy requirement, the robot will be adjusted to the desired attitude until the accuracy requirement is satisfied. After that, the robot starts to drill and countersink.

## 3. Normal Sensors Calibration

### 3.1. Measurement Model

Laser displacement sensors are commonly used to measure the surface normal for the robotic drilling system in industrial application. The points of a small area on workpiece surface are measured by three or more laser displacement sensors, and then the small area is approximately regarded as a micro-plane or micro-sphere and is fitted by using the fitting algorithm [13,15]. Finally, the surface normal at drilling point can be computed according to the fitted small area.

**Figure 3:**
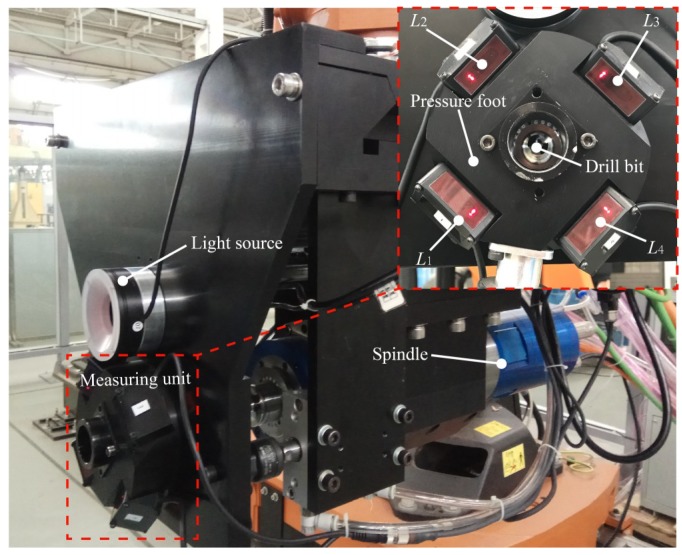
The installation of four laser displacement sensors.

As shown in Figure 3, *L_i_* (*i* = 1, 2, 3, 4) are respectively four laser displacement sensors. Four laser displacement sensors are uniformly installed on the pressure foot. The projection points of the laser beams emitted by sensors on the workpiece surface are within a small area to ensure the fitting accuracy of the small area.

The normal sensor calibration process will acquire the zero point and laser beam direction parameters of laser displacement sensors which describe the measurement process. After calibration, the surface normal measurement accuracy can be improved. The contribution of this section is constructing the measurement model and formulating the sensor parameter identification.

**Figure 4:**
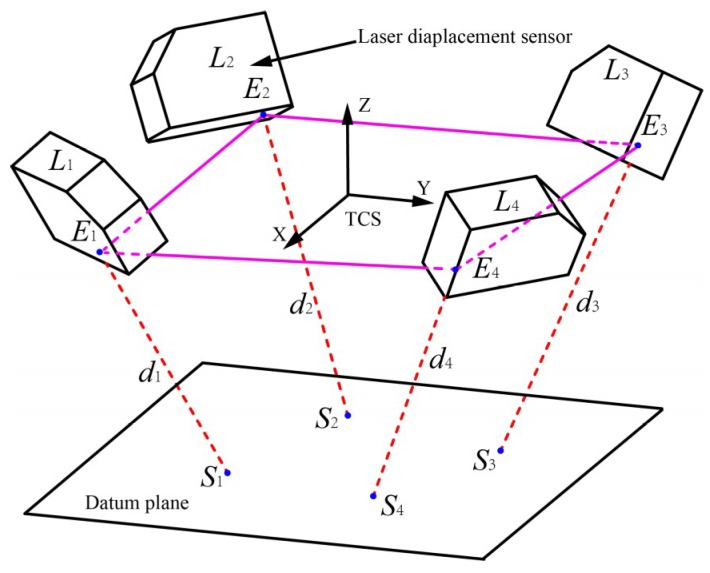
The measurement model of laser displacement sensors.

As shown in Figure 4, *L_i_* (*i* = 1, 2, 3, 4) are respectively four laser displacement sensors in the tool coordinate system (TCS) which is established by using laser tracker generally. *E_i_* (*i* = 1, 2, 3, 4) represent the zero points of four laser displacement sensors. The four projection points of the laser beams emitted by the laser displacement sensors on the datum plane are *S_i_* (*i* = 1, 2, 3, 4). *d_i_* (*i* = 1, 2, 3, 4) are the distances between the zero points and the corresponding projection points. Denoting the nominal zero point and laser beam directions of four laser displacement sensors in TCS as ${Ф}_{i}^{\mathrm{nom}}$ = [*x_i_*, *y_i_*, *z_i_*, *m_i_*, *n_i_*, *q_i_*]*^T^*, *i* = 1, 2, 3, 4.

Assuming that the equation of the datum plane in TCS is:

(1) Ax+By+Cz+D=0

where *A*, *B*, *C*, *D* are the parameters of datum plane. The equation of the datum plane can be calculated by fitting point cloud data measured by laser trackers [21].

The lines coinciding with the laser beams in TCS can be expressed as:

(2) $$\frac{x-x_{i}}{m_{i}}=\frac{y-y_{i}}{n_{i}}=\frac{z-z_{i}}{q_{i}}$$

Accordingly, the intersection points *S_i_* (*i* = 1, 2, 3, 4) of the lines and the datum plane can be obtained by solving Equations (1) and (2). Let [*x_si_*, *y_si_*, *z_si_*] be the coordinate values of intersection points *S_i_*. Then the *d_i_* between the zero points *E_i_* and the intersection points *S_i_* can be expressed as:

(3) $$d_{i}=\sqrt{{\left(x_{i}-x_{si}\right)}^{2}+{\left(y_{i}-y_{si}\right)}^{2}+{\left(z_{i}-z_{si}\right)}^{2}}$$

Equation (3) can also be written as follows:

(4) $$d_{i}=f(x_{i},y_{i},z_{i},m_{i},n_{i},q_{i})$$

Equation (4) shows that the theoretical measurement distances *d_i_* of the laser displacement sensors in TCS is related to the values of their zero points and laser beam directions. Due to the machining error of the pressure foot and the assembly error, there are small errors between the nominal and actual zero points and laser beam directions of laser displacement sensors. Denoting the errors of zero points and laser beam directions as Δ*x_i_*, Δ*y_i_*, Δ*z_i_*, Δ*m_i_*, Δ*n_i_*, Δ*q_i_*. Hence, the actual measurement distances of the laser displacement sensors are:

(5) $$d_{i}^{\prime }=f(x_{i}+\Delta x_{i},y_{i}+\Delta y_{i},z_{i}+\Delta z_{i},m_{i}+\Delta m_{i},n_{i}+\Delta n_{i},q_{i}+\Delta q_{i})$$

The measurement distance errors Δ*d_i_* can be expressed as:

(6) $$\Delta d_{i}=d_{i}^{\prime }-d_{i}$$

The total differential equation of Equation (6) can be written in the following form:

(7) $$\Delta d_{i}=\frac{\partial f}{\partial x_{i}}\Delta x_{i}+\frac{\partial f}{\partial y_{i}}\Delta y_{i}+\frac{\partial f}{\partial z_{i}}\Delta z_{i}+\frac{\partial f}{\partial m_{i}}\Delta m_{i}+\frac{\partial f}{\partial n_{i}}\Delta n_{i}+\frac{\partial f}{\partial q_{i}}\Delta q_{i}$$

or simply:

(8) $$\Delta d_{i}=J\Delta \Phi_{i}$$

where:

(9) $$J=\left[\frac{\partial f}{\partial x_{i}},\frac{\partial f}{\partial y_{i}},\frac{\partial f}{\partial z_{i}},\frac{\partial f}{\partial m_{i}},\frac{\partial f}{\partial n_{i}},\frac{\partial f}{\partial q_{i}}\right],\Delta \Phi_{i}={\left[\Delta x_{i},\Delta y_{i},\Delta z_{i},\Delta m_{i},\Delta n_{i},\Delta q_{i}\right]}^{T}$$

In Equation (8), six sensor parameter errors ΔФ*_i_* for each laser displacement sensor *L_i_* should be identified.

### 3.2. Parameter Identification with EKF Algorithm

EKF is an important optimization algorithm to estimate the optimal state [22]. The EKF has the advantage of reliability and fast convergence. It has been applied in many fields, such as simultaneous localization and mapping [23], tracking [24], parameter identification [25,26]. A parameter identification method based on EKF is applied to estimate the kinematic parameter errors of robot [27]. Nguyen et al. presented a calibration method using EKF to estimate the geometric parameters of industrial robot [25,26].

Denoting the vector of sensor parameter errors *X* = ΔФ*_i_* as the vector of state variables. Since the sensor parameter errors ΔФ*_i_* are all constant, the system equation of an EKF for sensor parameter identification can be formatted as:

(10) $$X_{k}=X_{k-1}+w_{k-1}$$

where *X_k_* is a 6-by-1 vector representing the sensor parameter errors at time step *t_k_*. *w_k_*_−1_ is a 6-by-1 vector denoting the process noise which is Gaussian white noise with zero mean. *Q_k_* is a 6-by-6 covariance matrix of the vector *w_k_*_−1_.

To identify the sensor parameters vector X, EKF algorithm needs measurement from the TCS and datum plane. A laser tracker is used to construct TCS firstly. The point cloud data on the datum plane measured by using laser tracker in TCS are fitted to achieve the equation of the datum plane. The equation of lines coinciding with the laser beams can be computed according to the nominal zero point and laser beam direction. Then the intersection point values can be obtained. The distances between the intersection points and the zero points are the nominal measurement distances $d_{i}^{\mathrm{nom}}$ of laser displacement sensors, which are recorded. The laser displacement sensors readings are also recorded simultaneously, which are defined as $d_{i}^{\mathrm{meas}}$. Then the measurement distance errors at time step *t_k_* can be calculated by Δ*d_i,k_* = $d_{i,k}^{\mathrm{meas}}$ − $d_{i,k}^{\mathrm{nom}}$ = *J_k_*ΔФ*_i_* (from Equation (8)). The measurement equation of EKF can be expressed as:

(11) $$Z_{k}=H_{k}X_{k}+v_{k}$$

where *Z_k_* = Δ*d_k_* is a 1-by-1 vector standing for the measurement distance error at time step *t_k_*. $H_{k}=J{|}_{{\hat{d}}_{i,k|k-1}}$ denotes a 1-by-6 matrix which is calculated according to the Equation (9). *v_k_* is a 1-by-1 vector denoting the measurement noise which is Gaussian white noise with zero mean. *R_k_* is a 1-by-1 covariance matrix of the vector *v_k_*.

The EKF algorithm consists of two steps: prediction and update. In the prediction step for EKF, predicted state can be expressed as:

(12) $${\hat{X}}_{k|k-1}={\hat{X}}_{k-1|k-1}$$

Predicted covariance is computed by:

(13) $$P_{k|k-1}=P_{k-1|k-1}+Q_{k-1}$$

In the update step for EKF, the optimal Kalman gain is:

(14) $$K_{k}=P_{k|k-1}H_{k}^{T}{\left(H_{k}P_{k|k-1}H_{k}^{T}+R_{k}\right)}^{-1}$$

Updated estimate covariance can be calculated by:

(15) $$P_{k|k}=(I-K_{k}H_{k})P_{k|k-1}$$

The estimated state is:

(16) $${\hat{X}}_{k|k}={\hat{X}}_{k|k-1}+K_{k}(Z_{k}-H_{k}{\hat{X}}_{k|k-1})$$

The initial value of the sensor parameter errors is ${\hat{X}}_{0}=0$. The matrix *Q* is initialized with 10^−10^ × ***I*** and *R* is 10^−4^ × ***I***. The initial value of matrix *P_0_* is ***I***. When EKF algorithm meets the stopping condition $\left\|{\hat{X}}_{k|k}-{\hat{X}}_{k-1|k-1}\right\|<\delta$, the parameter identification process is stopped. δ is 10^−4^ in this paper.

The solution of EKF algorithm is the sensor parameter errors ΔФ*_i_*. The modified sensor parameters ${Ф}_{i}^{\mathrm{mod}}$ = [$x_{i}^{\mathrm{mod}}$, $y_{i}^{\mathrm{mod}}$, $z_{i}^{\mathrm{mod}}$, $m_{i}^{\mathrm{mod}}$, $n_{i}^{\mathrm{mod}}$, $q_{i}^{\mathrm{mod}}$]*^T^* can be calculated by ${Ф}_{i}^{\mathrm{mod}}$ = ${Ф}_{i}^{\mathrm{nom}}$ + ΔФ*_i_*, where ${Ф}_{i}^{\mathrm{nom}}$ is the nominal sensor parameters.

## 4. Normal Adjustment

### 4.1. Surface Normal Measurement

**Figure 5:**
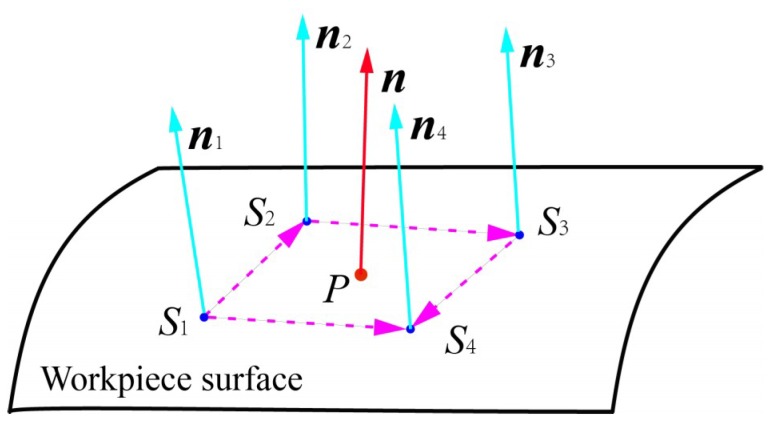
The surface normal measurement.

The modified sensor parameters in TCS are computed in Section 3.2. As shown in Figure 5, the distance *d_i_* between the sensors zero points *E_i_* (*i* = 1, 2, 3, 4) and the projection points *S_i_* (*i* = 1, 2, 3, 4) on the workpiece surface can be measured by the laser displacement sensors. Then the coordinate value of projection points on the workpiece in TCS can be calculated by:

(17) $$\left[\begin{array}{c} x_{si}\\ y_{si}\\ z_{si} \end{array}\right]=\left[\begin{array}{c} x_{i}^{\mathrm{mod}}\\ y_{i}^{\mathrm{mod}}\\ z_{i}^{\mathrm{mod}} \end{array}\right]+d_{i}\left[\begin{array}{c} m_{i}^{\mathrm{mod}}\\ n_{i}^{\mathrm{mod}}\\ q_{i}^{\mathrm{mod}} \end{array}\right]$$

The micro-plane method is one of most used surface normal measurement methods [28]. In this method, the micro workpiece surface near the drilling point is considered to be a micro-plane and the surface normal ***n*** at drilling point *P* can be approximately equal to the normal ***n****_i_* at projection points *S_i_*. Hence the normal ***n****_i_* at projection points *S_i_* can be expressed as:

(18) $$\left\{ \begin{array}{l} n_{1}=\frac{\vec{S_{1}S_{2}}\times \vec{S_{1}S_{4}}}{\left\|\vec{S_{1}S_{2}}\times \vec{S_{1}S_{4}}\right\|}\\ n_{2}=\frac{\vec{S_{1}S_{2}}\times \vec{S_{2}S_{3}}}{\left\|\vec{S_{1}S_{2}}\times \vec{S_{2}S_{3}}\right\|}\\ n_{3}=\frac{\vec{S_{2}S_{3}}\times \vec{S_{3}S_{4}}}{\left\|\vec{S_{2}S_{3}}\times \vec{S_{3}S_{4}}\right\|}\\ n_{4}=\frac{\vec{S_{1}S_{4}}\times \vec{S_{3}S_{4}}}{\left\|\vec{S_{1}S_{4}}\times \vec{S_{3}S_{4}}\right\|} \end{array}\right.$$

Then, the surface normal at drilling point *P* can be expressed as the weighted average of the normal ***n****_i_* at projection points *S_i_*, as shown in Equation (19):

(19) $$n=\frac{1}{4}\sum_{i=1}^{4}n_{i}$$

### 4.2. Attitude Adjustment

**Figure 6:**
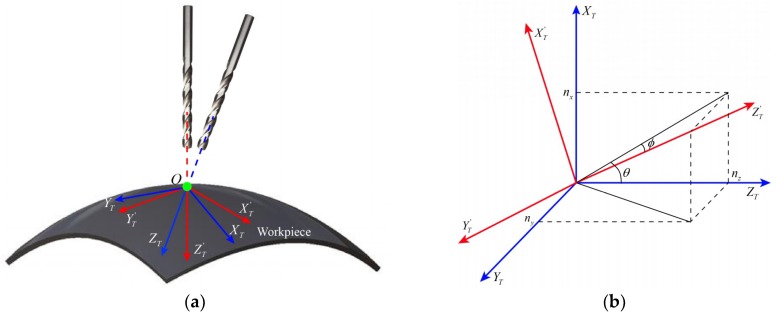
(**a**) The diagram of attitude adjustment. (**b**) Surface normal vector decomposition in TCS.

To ensure the perpendicularity accuracy of holes, the axis of the drill bit should be adjusted to coincide with the surface normal at the drilling point, as shown in Figure 6. The feed direction of drill bit is the positive z-axis of the TCS. Denoting the unit vector of drill bit in TCS before adjustment as ***e****_z_* = [0, 0, 1]*^T^*. After attitude adjustment, the positive z-axis of the TCS coincides with the surface normal ***n*** = [*n_x_*, *n_y_*, *n_z_*]*^T^* at drilling point. The attitude adjustment process of the drill bit is essentially the coordinate transformation of the TCS, i.e., the transformation from $OX_{T}Y_{T}Z_{T}$ to $OX_{T}^{\prime }Y_{T}^{\prime }Z_{T}^{\prime }$ shown in Figure 6. Firstly, the drill bit rotates *φ* degree around the Z-axis of TCS, and then rotates *θ* degree around the Y-axis of TCS. Finally, the drill bit rotates ϕ degree around the X-axis of TCS. The coordinate transformation can be expressed as:

(20) $$n=Rot(Z,\varphi )Rot(Y,\theta )Rot(X,\phi )e_{z}$$

where:

(21) $$Rot(Z,\varphi )=\left[\begin{array}{ccc} \cos\varphi & -\sin\varphi & 0\\ \sin\varphi & \cos\varphi & 0\\ 0 & 0 & 1 \end{array}\right],\;Rot(Y,\theta )=\left[\begin{array}{ccc} \cos\theta & 0 & \sin\theta \\ 0 & 1 & 0\\ -\sin\theta & 0 & \cos\theta \end{array}\right],\;Rot(X,\phi )=\left[\begin{array}{ccc} 1 & 0 & 0\\ 0 & \cos\phi & -\sin\phi \\ 0 & \sin\phi & \cos\phi \end{array}\right]$$

The angular of coordinate transformation can be computed by the geometric relationships in Figure 6. The computed results are:

(22) $$\left\{ \begin{array}{l} \varphi =0\\ \theta =\tan^{-1}(n_{x}/n_{z})\\ \phi =-\sin^{-1}(n_{y}) \end{array}\right.$$

where, $\theta ,\phi \in (-\frac{\pi }{2},\frac{\pi }{2})$.

Because of the measurement errors of laser displacement sensors and motion errors of drill bit, a one-time attitude adjustment may not meet the accuracy requirement, hence, the drill bit is adjusted until the angular deviation meets the requirement.

## 5. Simulation and Results

**Figure 7:**
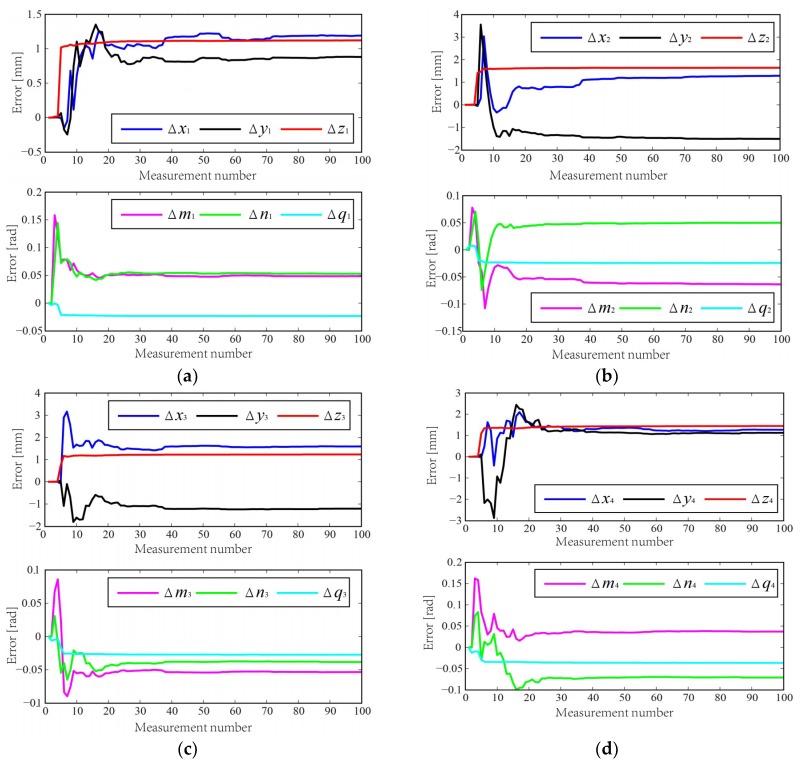
Estimated parameter errors of four laser displacement sensors using EKF algorithm.

To verify the correctness and validity of the proposed calibration method, a simulation is designed for normal sensor calibration. The simulation process is depicted as follows:

1. A datum plane is chosen, and its parameters are shown in Table 1. Twenty random points on the datum plane are selected, then the 20 points are added random measurement errors with a normal distribution N (0, ξ), where the standard deviation ξ is 0.02 mm. The equation of fitted planed can be computed by using the method in [21].
2. An appropriate TCS is constructed. The assumptive nominal sensor parameters and corresponding parameter errors in TCS are shown in Table 2.
3. According to the nominal sensor parameter, the theoretical measurement model is established using the method in Section 3.1. Hence the theoretical measurement distance *d_i_* can be obtained.
4. The sensor parameter errors are added to the nominal sensor parameters. The actual measurement model can be also established by using method in Section 3.1. The actual measurement distance $d_{i}^{\prime }$ is computed and then it is added with a normal distribution N (0, ζ), where the standard deviation ζ is 0.01 mm.
5. The measurement distance errors Δ*d_i_* can be calculated by Equation (6).
6. Then posture of TCS is changed, and the operation in Step 2–5 is repeated again 99 times.
7. The sensor parameter errors can be identified by using EKF algorithm in Section 3.2. The matrix Q is 10^−10^ × ***I*** and *R* is 8 × 10^−5^ × ***I***. The estimated sensor parameter errors are shown in Figure 7. The modified sensor parameters can be calculated.
8. A plane is chosen as the workpiece surface and the normal of the plane is recorded. An appropriate TCS is constructed. Repeat the operation in Step 4 to obtain the actual measurement distance $d_{i}^{\prime }$.
9. The normal at drilling robot before and after calibration can be computed based on the nominal and modified sensor parameters by using the method in Section 4.1, respectively. Therefore, the angular deviation of surface normal measurement before and after calibration can be obtained.
10. Change the posture of the plane. Repeat the operation of Steps 8~9 again 19 times.

**Figure 8:**
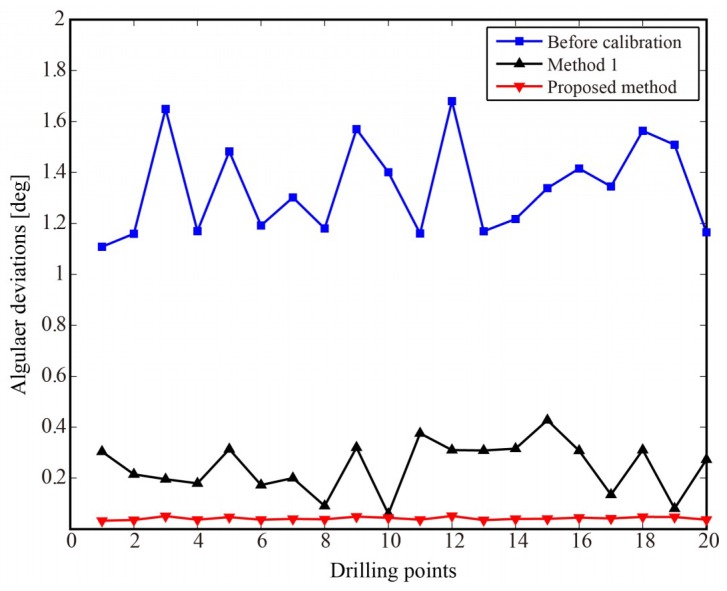
The angular deviation after calibration of the methods.

Table 1 presents that the fitted plane mostly coincides with the theoretical plane. Hence, the high fitting precision method provides a guarantee for the calibration accuracy of laser displacement sensors. As shown in Table 3, the numbers of convergence measurement points for each laser displacement sensor are almost same. The calibration results by using proposed method are compared with that by using the method from [19] which is referred to as method 1. In method 1, the position and orientation of laser displacement sensors were identified by using nonlinear least squares method based on the model of plane fitting. The simulation results are shown in Figure 8. Table 4 summarizes the statistics of the simulation results. After calibration, the average angular deviation of normal is improved significantly to 0.0412° from 1.3387°, and the maximum angular deviation of normal is also reduced to 0.0511° from 1.6801°.

The angular deviations of normal after calibration are obviously less than the required accuracy of 0.5° [9,29]. The simulation result shows that the proposed calibration method has higher accuracy than method 1 and can enhance the surface normal measurement accuracy and meet the requirement of robotic drilling. It also demonstrates the correctness and validity of the proposed calibration method.

## 6. Experiments and Results

**Figure 9:**
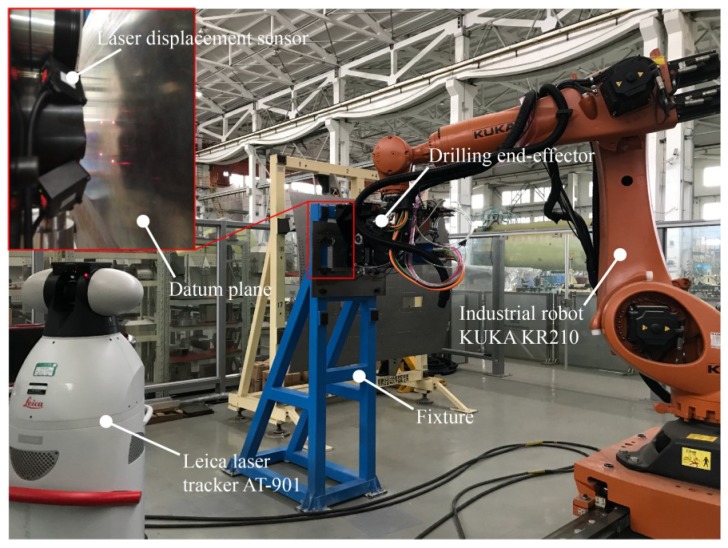
The experimental setup of the robotic drilling system.

**Figure 10:**
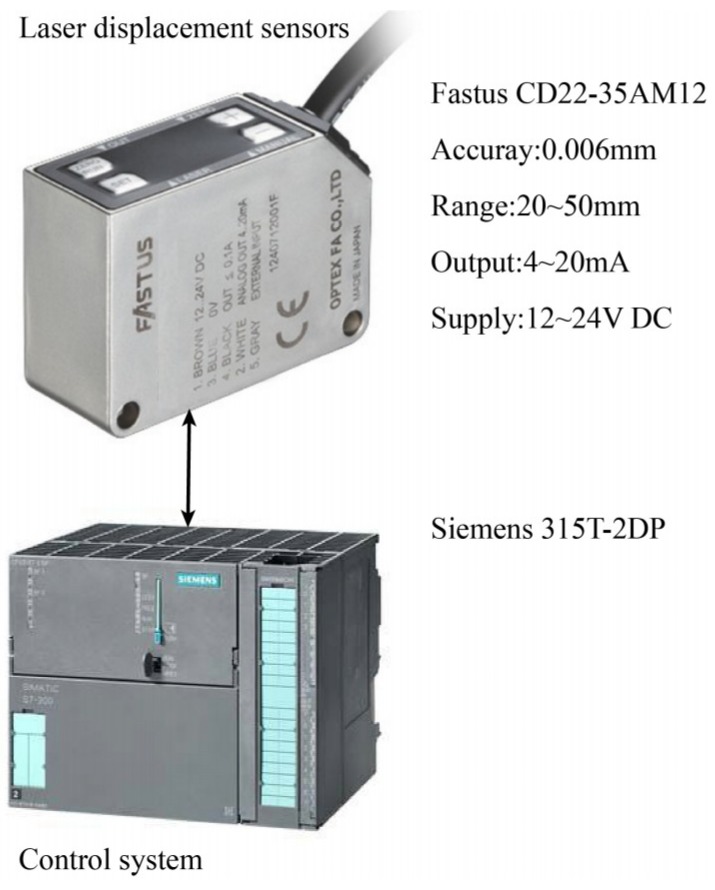
The hard structure of data collect system for laser displacement sensors.

To study the performance of the proposed calibration method, experiments are conducted on a robotic drilling system, as shown in Figure 9. The experimental setup for calibration consists of a KUKA KR210 industrial robot, drilling end-effector, Leica laser tracker AT-901, fixture, datum plane, laser tracker displacement sensors, etc. The absolute distance measurement accuracy of the laser tracker is 7.5 μm + 3 μm/m. The datum plane is installed on a fixture with high stiffness. The hard structure of data collect system of used laser displacement sensors is illustrated in Figure 10. The measured distances of laser displacement sensors are collected and converted to digital signals by using a Siemens 315T-2DP controller. The Siemens 315T-2DP controller can realize sensor data acquisition, motion control, data processing and other functions in robotic drilling.

**Figure 11:**
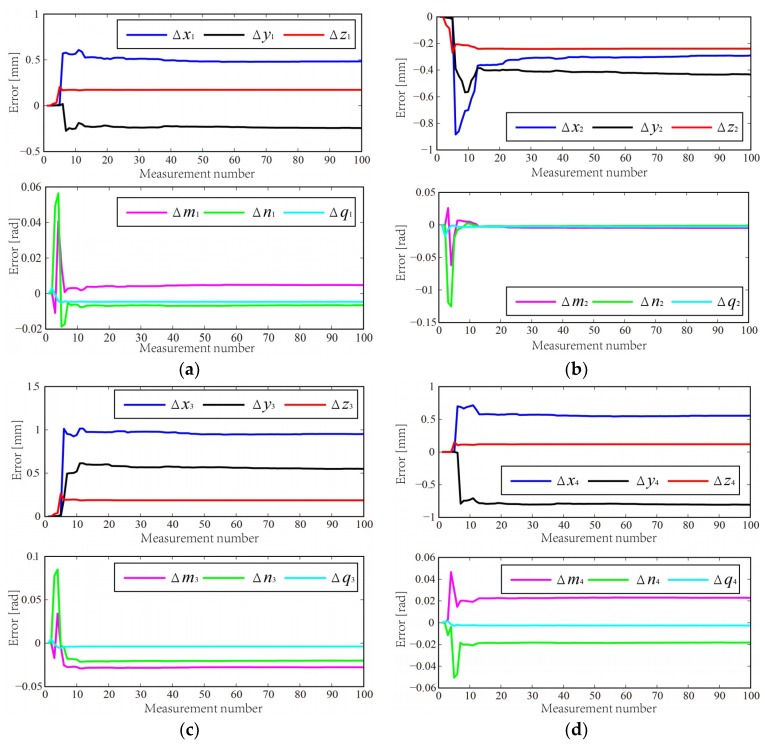
Estimated parameter errors of sensors using EKF algorithm.

As shown in Figure 9, the datum plane can be calculated by fitting the point cloud data measured by laser trackers. The parameters of the fitted datum plane in the laser tracker frame are shown in Table 5. The TCS is constructed in the laser tracker frame. The industrial robot moves the drilling end-effector to a suitable position so that the laser displacement sensors can measure the datum plane and the measurement distances are within the sensor range. The measured distances of sensors are recorded and the TCS in laser tracker frame is measured. The above action is repeated 99 times and then 100 sets of data are obtained. According to the collected data, the sensor parameter errors can be identified by using EKF algorithm. The matrix Q is 10^−12^ × ***I*** and *R* is 9 × 10^−6^ × ***I***. Table 6 and Figure 11 show the estimated sensor parameter errors. The modified sensor parameters can be computed.

**Figure 12:**
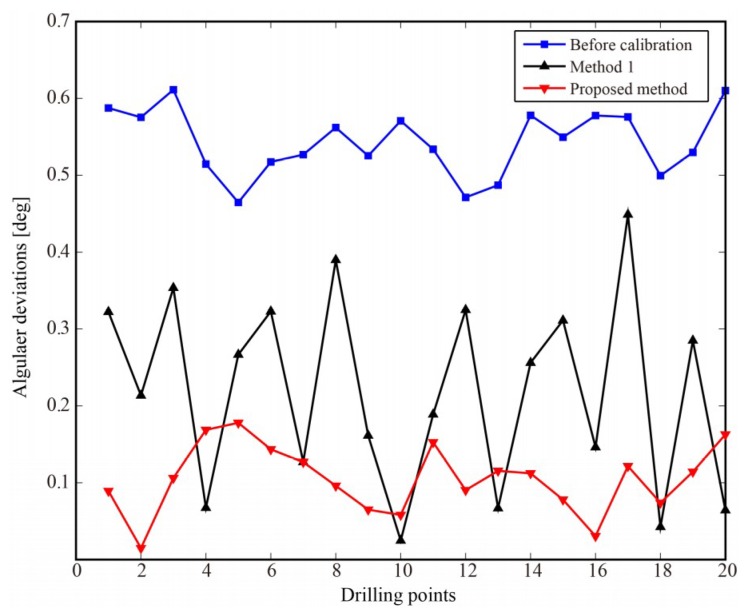
The angular deviation after experimental calibration by the methods.

As shown in Table 7, the numbers of convergence measurement points for each laser displacement sensor are almost same in the experiment. Normal measurement and attitude adjustment are performed using nominal sensor parameters and modified sensor parameters, respectively. The angles between the normal of the datum plane and the feed direction of spindle can be measured by the laser tracker. The angular deviation after experimental calibration by the methods is shown in Figure 12. Table 8 summarizes the statistics of the angular deviation after experimental calibration by the methods. 

The average and maximum angular deviation after calibration are all less than that before calibration. The average angular deviation is 0.1048° with a maximum value of 0.1780°. Table 6 shows that the angular deviations are all less than the required accuracy of 0.5° and the proposed calibration method has higher accuracy than method 1. The calibration and experimental results show that the proposed calibration method can improve the perpendicularity accuracy of drilling and meet the accuracy requirement of aircraft assembly. The experimental results also demonstrate correctness and validity of the proposed calibration method.

## 7. Conclusions

This paper presented a normal sensor calibration method to improve the perpendicularity accuracy in a robotic drilling system. The measurement model based on the principle of the distance measurement is constructed and the sensor parameter errors are identified by using the EKF algorithm. The simulation results demonstrate the correctness and validity of the proposed calibration method. The calibration experiment is performed on the robotic drilling system. With the proposed calibration method, the average angular deviation is reduced to 0.1048° from 0.5434° and the maximum angular deviation is reduced to 0.1780° from 0.6113°. In addition, the experimental results show that the calibration method can enhance the perpendicularity accuracy of drilling and meet the accuracy requirements of aircraft assembly.

## Figures and Tables

**Table 1:** The parameters of the simulated plane.

Parameters	A	B	C	D
Theoretical plane	0.0173	−0.3071	0.9515	34.1218
Fitted plane	0.0173	−0.3070	0.9516	34.1285

**Table 2:** The assumptive nominal sensor parameters and corresponding parameter errors.

*L_i_*	*x_i_* + Δ*x_i_* [mm]	*y_i_* + Δ*y_i_* [mm]	*z_i_* + Δ*z_i_* [mm]	*m_i_* + Δ*m_i_* [rad]	*n_i_* + Δ*n_i_* [rad]	*q_i_* + Δ*q_i_* [rad]
1	50.0000 + 1.3000	0.0000 + 0.9000	0.0000 + 1.1000	−0.5000 + 0.0445	0.0000 + 0.0506	−0.8660 − 0.0228
2	0.0000 + 1.3000	50.0000 − 1.4000	0.0000 + 1.6000	0.0000 − 0.0634	−0.5000 + 0.0479	−0.8660 − 0.0237
3	−50.0000 + 1.3000	0.0000 − 1.2000	0.0000 + 1.2000	0.5000 − 0.0507	0.0000 − 0.0365	−0.8660 − 0.0266
4	0.0000 + 1.5000	−50.0000 + 1.1000	0.0000 + 1.4000	0.0000 + 0.0339	0.5000 − 0.0682	−0.8660 − 0.0353

**Table 3:** The numbers of convergence measurement points for each laser.

	*L*_1_	*L*_2_	*L*_3_	*L*_4_
Number	75	86	91	81

**Table 4:** The statistics of the angular deviation after calibration of the methods.

	Max. [deg]	Mean [deg]	Std. [deg]
Before	1.6801	1.3387	0.1841
Method 1	0.4278	0.2446	0.1024
Proposed method	0.0511	0.0412	0.0057

**Table 5:** The parameters of fitted datum plane.

Parameters	A	B	C	D
Fitted plane	0.4497	0.8932	−0.0084	−1690.2827

**Table 6:** The nominal sensor parameters and estimated sensor parameter errors using EKF algorithm.

*L_i_*	*x_i_* + Δ*x_i_* [mm]	*y_i_* + Δ*y_i_* [mm]	*z_i_* + Δ*z_i_* [mm]	*m_i_* + Δ*m_i_* [rad]	*n_i_* + Δ*n_i_* [rad]	*q_i_* + Δ*q_i_* [rad]
1	−36.4655 + 0.4838	36.4655 − 0.2460	−35.0000 + 0.1723	0.3536 + 0.0047	−0.3536 − 0.0065	0.8660 − 0.0047
2	36.4655 − 0.2906	36.4655 − 0.4338	−35.0000 − 0.2393	−0.3536 − 0.0048	−0.3536 − 0.0012	0.8660 − 0.0026
3	36.4655 + 0.9513	−36.4655 + 0.5488	−35.0000 + 0.1860	−0.3536 − 0.0278	0.3536 − 0.0201	0.8660 − 0.0039
4	−36.4655 + 0.5558	−36.4655 − 0.8081	−35.0000 + 0.1186	0.3536 + 0.0229	0.3536 − 0.0182	0.8660 − 0.0026

**Table 7:** The numbers of convergence measurement points for each laser.

	*L*_1_	*L*_2_	*L*_3_	*L*_4_
Number	39	44	34	44

**Table 8:** The statistics of the angular deviation after experimental calibration by the methods.

	Max. [deg]	Mean [deg]	Std. [deg]
Before	0.6113	0.5434	0.0432
Method 1	0.4487	0.2192	0.1270
Proposed method	0.1780	0.1048	0.0443
